# Arsenic trioxide-induced acute kidney injury: OPA1- and Drp1-mediated mitochondrial dynamics imbalance, PINK1/Parkin-dependent mitophagy, and Chuanhuang Fang III

**DOI:** 10.3389/fmolb.2026.1778855

**Published:** 2026-01-30

**Authors:** Peiji Wang, Zicong Wu, Zhiyong Song, Xingyu Deng, Yifan Zhang, Xuezhong Gong

**Affiliations:** Department of Nephrology, Shanghai Municipal Hospital of Traditional Chinese Medicine, Shanghai University of Traditional Chinese Medicine, Shanghai, China

**Keywords:** acute kidney injury, arsenic trioxide, Chuanhuang Fang III, mitochondrial quality control, mitochondrialdynamics, mitophagy

## Abstract

**Purpose:**

Acute kidney injury (AKI) remains a global health concern with limited therapies. Among its causes, arsenic (AS)-induced AKI (AI-AKI), exemplified by the antitumor agent arsenic trioxide (ATO), represents an emerging clinical challenge. Despite its clinical efficacy in treating AI-AKI, the protective mechanism of Chuanhuang Fang III (CHF) remains unclear. This study aimed to investigate the mechanisms and therapeutic targets of CHF against AI-AKI.

**Methods:**

Classic AI-AKI rat model was established, and subsequently treated with graded doses of CHF. CHF constituents were identified. Renal pathology, renal function, and AKI biomarkers were detected. Mitochondrial quality control-related parameters were detected as follows: 1) transmission electron microscopy was employed to assess mitophagy; 2) Western blotting was performed to evaluate mitochondrial dynamics- and mitophagy-related proteins, while differential gene expression and pathway enrichment were analyzed by RNA-sequencing; 3) mitochondrial membrane potential and mitochondrial ROS levels were measured in freshly isolated renal cortical mitochondria by JC-1 staining and flow cytometry. The HK-2 cell line was used to further elucidate the underlying mechanisms of AI-AKI, and the effect of antioxidant NAC was observed simultaneously.

**Results:**

ATO exposure resulted in increased serum creatinine, mitochondrial dysfunction, elevated mitochondrial ROS levels, and promoted apoptosis, autophagy, and mitophagy in renal tubular epithelial cells. It also downregulated the mitochondrial fusion protein OPA1 and upregulated the fission protein Drp1. These effects correlated with the activation of the PINK1/Parkin mitophagy pathway, as well as increased expression of BNIP3, NIX, LC3B and Bax, and decreased anti-apoptotic protein Bcl-2. Transcriptomic analysis indicated that the key signaling pathways in AI-AKI were associated with mitophagy, autophagy, mitochondrial function and apoptosis. CHF attenuated AI-AKI by regulating OPA1/Drp1 balance and PINK1/Parkin-mediated mitophagy and counteracted the associated pathological processes. *In vitro* experiments using the HK-2 cell line provided further evidence supporting the *in vivo* findings.

**Conclusion:**

The pathogenesis of clinical-dose ATO-induced AKI involves OPA1- and Drp1-mediated mitochondrial dynamics imbalance and PINK1/Parkin-dependent mitophagy in renal tubular epithelial cells, CHF ameliorated this injury by restoring mitochondrial quality control, highlighting its therapeutic potential against AI-AKI.

## 1 Introduction

Acute kidney injury (AKI) remains a significant health issue that urgently needs to be addressed worldwide, affecting approximately 13.3 million people each year and causing nearly two million deaths (Ostermann et al., 2025). Its main characteristics are rapid deterioration of renal function, accompanied by high morbidity and mortality rates, which impose a huge burden on public health (Chen et al., 2025). Among the various causes of AKI, exposure to environmental toxins, especially arsenic (AS) intake, has been confirmed as a key factor in inducing kidney damage (Hsu et al., 2025; Kingdom et al., 2025; Zhou et al., 2025). Arsenic trioxide (ATO) has been widely used in the standard treatment of acute promyelocytic leukemia (APL) (Yilmaz et al., 2021). However, an increasing amount of clinical evidence indicates that ATO can cause arsenic-induced AKI (AI-AKI) (Jen et al., 2024; Kong et al., 2025; Gao et al., 2024). A 12-year real-world study report shows that the incidence of AI-AKI in patients with acute promyelocytic leukemia (APL) is 4.5%, and this injury is directly related to the prognosis of patients in each risk group (Shen et al., 2025). Although these findings have made significant progress in clarifying the mechanism of arsenic-induced nephrotoxicity, the clinical preventive drug intervention regimens for AI-AKI are still relatively limited. Therefore, it is particularly important to explore the pathogenesis of AI-AKI and search for new preventive drugs.

Mitochondrial quality control (MQC) is essential for maintaining mitochondrial homeostasis and cellular viability. It encompasses multiple coordinated processes, including the regulation of mitochondrial dynamics, removal of damaged mitochondria, scavenging of reactive oxygen species (ROS), and maintenance of mitochondrial membrane potential. Disruption of MQC can result in mitochondrial dysfunction, ultimately leading to cellular injury. Among these processes, mitochondrial dynamics play a pivotal role in MQC (Sabouny and Shutt, 2020). Mitochondrial fusion facilitates the exchange of components between damaged and healthy mitochondria, thereby maintaining mitochondrial function, whereas mitochondrial fission isolates damaged segments that are selectively degraded through mitophagy, primarily mediated by the canonical PINK1/Parkin pathway (Ye et al., 2025; Burman et al., 2017). Optic atrophy type1(OPA1) preserves mitochondrial connectivity and cristae structure, whereas dynamin-related protein 1 (Drp1) mediates mitochondrial fission (Tábara et al., 2025). Excessive fusion produces an overly connected mitochondrial network that enhances ATP generation and reduces ROS, while excessive fission causes mitochondrial fragmentation and dysfunction (Li G. et al., 2025).

Chuanhuang Fang III (CHF) is an effective formula for treating AKI caused by nephrotoxic drugs at Shanghai Municipal Hospital of Traditional Chinese Medicine. Based on the results of multi-center randomized controlled clinical trials, Chuanhuang Fang has shown good efficacy in treating AKI in several hospitals in China (Chen et al., 2022; Chen and Gong, 2022). Our previous studies have shown this formula exerts renoprotective effects by reducing Serum creatinine (Scr), high-sensitivity C-reactive protein (hs-CRP), and AKI biomarkers in AKI patients (Gong et al., 2015; Song et al., 2023; Chen et al., 2024). However, the underlying molecular mechanism of CHF against AI-AKI is still unclear.

This study proposes the following hypothesis: ATO may evoke MQC by regulating OPA1- and Drp1-mediated mitochondrial dynamics and PINK1/Parkin-mediated mitophagy, thereby contributing to the development of AI-AKI. To test this hypothesis, we evaluated the renal injury induced by ATO through both *in vivo* and *in vitro* models. Furthermore, our findings demonstrate that CHF protects against AI-AKI by modulating MQC to preserve mitochondrial homeostasis, providing mechanistic evidence supporting its therapeutic potential in AI-AKI.

## 2 Materials and methods

### 2.1 Drugs and reagents

ATO and N-acetylcysteine (NAC) were obtained from Sigma-Aldrich (St. Louis, MO, United States). JC-1 Mitochondrial Membrane Potential Detection Kit, HE-staining Kit, and Annexin V-FITC/PI apoptosis detection kits were purchased from Beyotime Biotechnology (Shanghai, China). The herbs used in CHF was obtained from the Shanghai Municipal Hospital of Traditional Chinese Medicine. The medicinal herbs were identified by hospital pharmacy pharmacists and meet the quality standards specified in the *Chinese Pharmacopoeia 2020*. CHF included prepared Da Huang (*Rheum officinale* Baill), Chuan Xiong (*Ligusticum chuanxiong* Hort), Huang Lian (*Coptis chinensis* Franch), Dang Shen (*Codonopsis pilosula* (Franch.) Nannf), etc.

### 2.2 Component identification of CHF

CHF preparation adhered to established protocols (Li et al., 2023), combined herbs underwent twice-repeated decoction in pure water, and then the filtrates were placed in a rotary evaporator. After rotation, the filtrates were concentrated to 2.25 g/mL with distilled water for *in vivo* experiments. The chemical constituents were analyzed using Ultra High Performance Liquid Chromatography-Quadrupole Exactive Orbitrap High Resolution Mass Spectrometry (UHPLC-Q Exactive Orbitrap-HRMS).

### 2.3 Experimental animals and treatment

A well-established rat model of AI-AKI was used. All research protocols for animal experiments were previously approved by Animal Welfare and Ethical Use Committee of the Shanghai Municipal Hospital of Traditional Chinese Medicine (approval number: 2024363, Approval Date: 15 October 2024) and followed the ARRIVE guidelines. Male SD rats, weighing 200–250 g and aged 8–10 weeks were obtained from the Shanghai Laboratory Animal Research Center (certificate number: 2020-009). The establishment of the AI-AKI model was based on previous literature (Song et al., 2025a). Initially, Rats were randomly assigned to different groups: controls (CON), rats injected with ATO, ATO rats treated with the low-dose, medium-dose, and high-dose CHF (ATO + CHF-L, ATO + CHF-M, ATO + CHF-H), rats treated with NAC and ATO (ATO + NAC). Rats in the CHF groups received CHF-L (2.005 g/kg), CHF-M (4.01 g/kg), and CHF-H (8.02 g/kg).

### 2.4 Biochemical assays

Scr and blood urea nitrogen (BUN) levels were measured using commercial assay kits (Nanjing Jiancheng Bioengineering Institute, China). Urinary AKI biomarkers, including urinary N-acetyl-glucosaminidase (UNAG) and urinary-glutamyl transpeptidase (UGGT), were evaluated with an enzyme-linked immunosorbent assay kit according to the manufacturer’s instructions (MedicalSystem, Ningbo, China).

### 2.5 Transcriptomics analyses

Total RNA was isolated from renal tissues and subjected to quantitative and integrity assessments to ensure sample quality. RNA sequencing libraries were prepared by reverse transcription to cDNA and PCR amplification. The high-throughput sequencing was conducted on the Illumina NovaSeq 6000 platform. After quality control and data filtering, bioinformatic analyses were performed to quantify gene expression levels and investigate potential molecular mechanisms. Transcript expression was quantified as FPKM values, and differentially expressed genes (DEGs) were identified using DESeq2 for group comparisons and edgeR for pairwise analyses, with thresholds of false discovery rate (FDR) <0.05 and |fold change| ≥2. The resulting DEGs were subsequently subjected to Gene Ontology (GO) functional and Kyoto Encyclopedia of Genes and Genomes (KEGG) pathway enrichment analyses. RNA sequencing was conducted by OE Biotech Co., Ltd. (Shanghai, China).

### 2.6 Histopathological evaluation

Kidney tissues were fixed in 4% paraformaldehyde, embedded in paraffin, and sectioned at 3 μm. Sections were stained with hematoxylin and eosin (H&E) and examined under a light microscope (Leica Microsystems, Germany). Tubular injury was assessed based on tubular dilation, cytoplasmic vacuolization, and epithelial detachment. Tubular injury score was estimated in renal tissue stained with hematoxylin and eosin, as described previously (Yang et al., 2018).

### 2.7 Transmission electron microscopy (TEM)

Fresh renal tissues were cut into 1 mm^3^ cubes and processed using standard histological steps as described in our previously published studies (Song et al., 2025a; Gong et al., 2019). Ultrastructural changes and mitophagy were examined and imaged using a transmission electron microscope (Hitachi, Tokyo, Japan).

### 2.8 Cell culture and treatment

Human renal proximal tubular epithelial cells (HK-2, American Type Culture Collection, Manassas, VA, United States) were cultured in DMEM/F12 medium containing 10% fetal bovine serum and 1% penicillin-streptomycin at 37 °C in a humidified atmosphere with 5% CO_2_. According to the previous method (Song et al., 2025a), the model group was treated with the corresponding dose of trivalent arsenic.

### 2.9 Preparation of rat kidney cortex mitochondria and mitochondrial membrane potential detection (ΔΨm)

Tissue Mitochondria Isolation Kit (Beyotime Biotechnology, China) was used to isolate intact mitochondria of the rat kidney according to the manufacturer’s instruction. In brief, after homogenization of fresh kidney tissue in ice-cold buffer provided with the kit, homogenized tissues were centrifuged at 6,000 g for 5 min at 4 °C. The supernatant was collected and further centrifuged at 1,100 g at 4 °C for 10 min to enrich the mitochondrial fraction for the measurement of membrane potential ΔΨm using the mitochondrial membrane potential assay kit with JC-1 dye (Beyotime Biotechnology, China). As a cationic dye, JC-1 aggregates in the mitochondrial matrix and emits red fluorescence at 590 nm, indicative of intact ΔΨm. Contrarily, when JC-1 exists in monomeric form and emits green fluorescence at 525 nm, the signal indicates apoptotic and dead cells. According to the protocol of the kit, the red/green-fluorescent intensity ratio at 590–525 nm as determined by immunofluorescence microscope was used to indicate the ΔΨm level.

### 2.10 Measurement of mitochondrial ROS production in rat kidney cortex

ROS production in the mitochondria of rat kidney cortex was measured by the fluorescent probe DCFH-DA (2, a7-Dichlorofuorescin Diacetate) immediately following mitochondria isolation as described above. Briefly, using the reactive oxygen species Assay Kit (Nanjing Jiancheng, China), isolated mitochondria were incubated with 10 μM DCFH-DA at 37 °C for 60 min, the fluorescence intensity of dichlorofluorescein (DCF) was measured at an excitation light wavelength of 490 nm and emission light wavelength of 525 nm by flow cytometry.

### 2.11 Apoptosis analysis

Cell apoptosis was analyzed using the Annexin V-FITC/PI kit (Beyotime Biotechnology, China). HK-2 cells were stained with Annexin V-FITC and PI for 15 min in the dark and analyzed by flow cytometry. The proportion of early and late apoptotic cells was quantified as the total apoptosis rate.

### 2.12 Immunofluorescence co-localization

HK-2 cells were prepared following standard procedures and then reacted with Parkin (Abcam, ab77924, 1:200) antibody at 4 °C overnight. On the next day, they were reacted with secondary antibody for 50 min, stained with TSA-488 working solution (G1236-1, Service-bio, China) for 10 min. After three washes with PBS buffer, they were reacted with OPA1 (Proteintech, 27733-1-AP, 1:200) antibody at 4 °C overnight. On the third day, they were incubated with secondary antibody, stained with TSA-555 working solution (G1236-2, Servicebio) and DAPI, finally visualized using a laser scanning confocal microscope.

### 2.13 Western blot analysis

Western blotting analysis for rat kidney cortex tissue was conducted as described previously (Gong et al., 2013). The primary antibodies used in the current study included: Parkin (1:1,000); PTEN-induced putative kinase 1 (PINK1, Proteintech, 23274-1-AP, 1:1,000); B-cell leukemia/lymphoma 2 (Bcl-2, Proteintech, 26593-1-AP, 1:1,000); Bcl-2-associated X protein (BAX, Abcam, ab32503, 1:1,000); Bcl-2 interacting protein 3(BNIP3, Abcam, ab109362, 1:1,000); NIP3-like protein X (NIX, Abcam, ab109414, 1:1,000); OPA1 (1:1,000); Drp1 (Proteintech, 12957-1-AP, 1:1,000); Microtubule-associated protein 1 light chain 3 beta (LC3, Proteintech, 14600-1-AP, 1:1,000); Glyceraldehyde-3-phosphate dehydrogenase (GAPDH, Proteintech, 60004-1-Ig, 1:1,000).

### 2.14 Statistical analysis

GraphPad Prism 10.0 software was utilized for the analysis of quantitative data. All experimental data were presented as mean ± SD and the differences among groups were assessed using one-way ANOVA. A *p*-value <0.05 was considered to indicate statistical significance.

## 3 Results

### 3.1 Identification of the components of CHF using UHPLC-Q Exactive Orbitrap-HRMS

**FIGURE 1:**
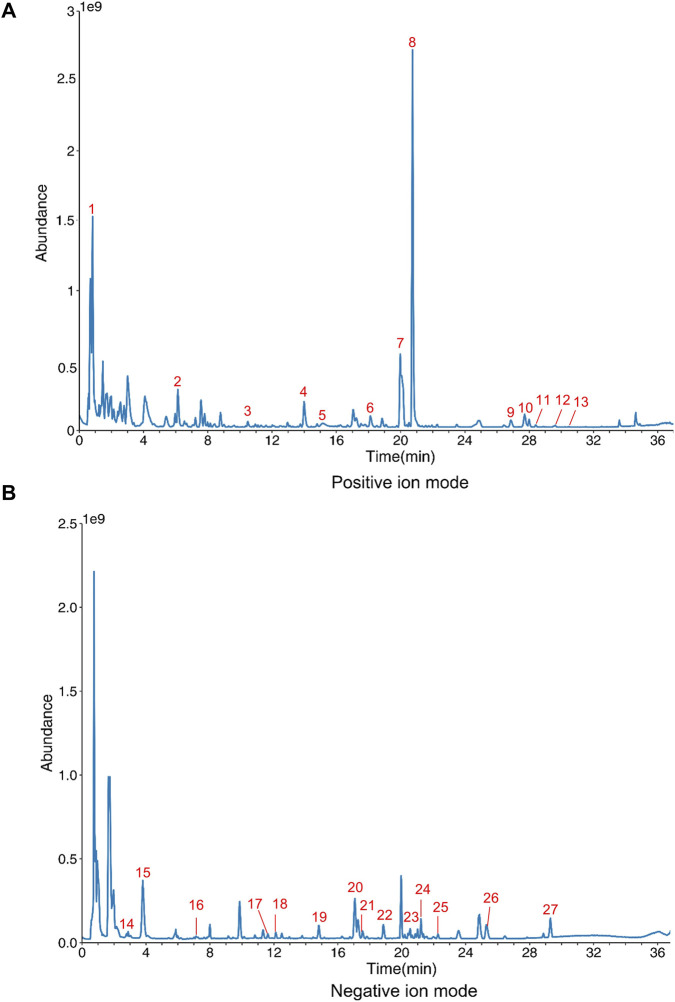
Component identification of CHF using UHPLC-Q Exactive Orbitrap-HRMS. **(A)** CHF sample under positive ion mode. **(B)** CHF sample under negative ion mode. (1. Trigonelline; 2. Cordycepin; 3. Ligustrazine; 4. Magnoflorine; 5. Syringaldehyde; 6. Coniferaldehyde; 7. Senkyunolide I; 8. Palmatine; 9. Scutellarein tetramethyl ether; 10. Senkyunolide A; 11. Tangeretin; 12. Ligustilide; 13. Atractylenolide II; 14. Arbutin; 15. Gallic acid; 16. Danshensu; 17. Chlorogenic acid; 18. Cryptochlorogenic acid; 19. 4-Feruloylquinic acid; 20. Vitexin; 21. Luteolin-7-rutinoside; 22. lsoacteoside; 23. lsoliquiritin; 24. Chrysophanol 8-O-glucoside; 25. Physcion 1-0-beta-D-glucoside; 26. Rhein; 27. Emodin).

To identify the main components of CHF, we analyzed its chemical composition using UHPLC-Q Exactive Orbitrap-HRMS. In total, 27 major compounds were identified. The chromatograms obtained in both positive and negative ion modes are shown in (Figure 1). The identified constituents included amino acids, alkaloids, phenols, flavonoids, and terpenes. These components comprise Trigonelline, Cordycepin, Ligustrazine, Magnoflorine, Syringaldehyde, Coniferaldehyde, Senkyunolide I, Palmatine, Scutellarein tetramethyl ether, Senkyunolide A, Tangeretin, Ligustilide, Atractylenolide II, Arbutin, Gallic acid, Danshensu, Chlorogenic acid, Cryptochlorogenic acid, 4-Feruloylquinic acid, Vitexin, Luteolin-7-rutinoside, lsoacteoside, lsoliquiritin, Chrysophanol 8-O-glucoside, Physcion 1-0-beta-D-glucoside, Rhein, and Emodin. Detailed information is provided in the Supplementary Material.

### 3.2 CHF alleviated kidney and mitochondrial damage in AI-AKI rat model

**FIGURE 2:**
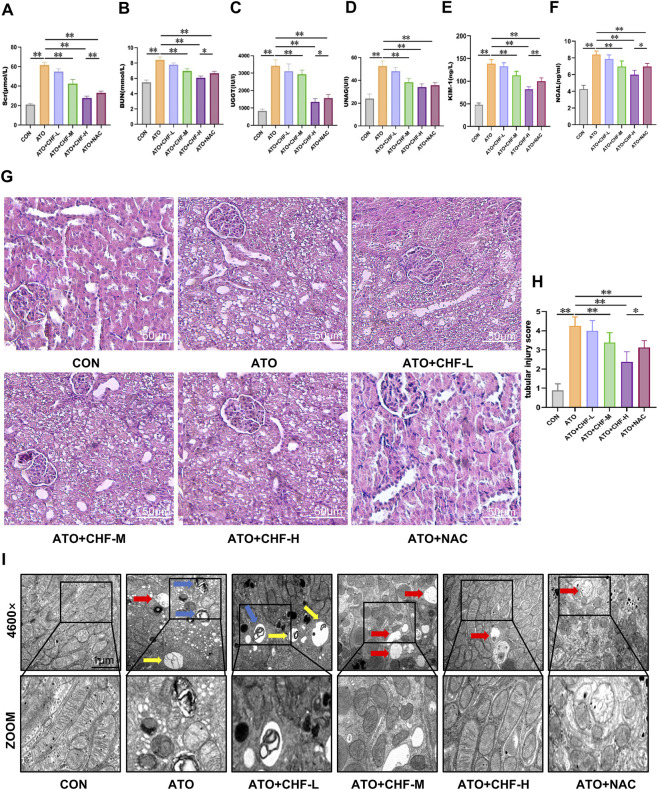
CHF alleviated kidney and mitochondrial damage in AI-AKI rat model. **(A–F)** Scr, BUN, UNAG, UGGT, KIM-1 and NGAL levels in rats. (*p < 0.05, **p < 0.01 vs. CON or ATO, n = 5) **(G)** H&E staining of kidney tissues from ATO rats (original magnification, ×200). **(H)** Mean tubular injury scores of ten randomly selected high-power fields in rat kidney sections. **(I)** Representative images of mitophagy in kidney tissues based on transmission electron microscopy (4,600×). Blue arrows indicate mitophagy, yellow arrows indicate autophagy, red arrows indicate swollen mitochondria er mitochondrial vacuolization.

To evaluate the renoprotective effect of CHF on AI-AKI, rats were pretreated with different doses of CHF. After exposure to ATO, the levels of Scr and BUN, and urinary AKI biomarkers UGGT and UNAG significantly increased, indicating that ATO caused acute renal function injury in rats and the AI-AKI model was successfully constructed. Additionally, AKI biomarkers KIM-1 and NGAL were also significantly elevated. After treating the models with different concentrations of CHF, it was found that the medium and high-dose groups of CHF could reduce the levels of Scr, BUN, UGGT and UNAG, among which the high-dose group showed the most significant renoprotective effect (Figures 2A–F). Interestingly, the results also indicated that the high-dose CHF group exhibited a more pronounced renoprotective effect compared with the NAC group.

Histopathological analysis showed that there was obvious swelling of renal tubular epithelium, cytoplasmic vacuolation and inflammatory infiltration in the ATO group. CHF can significantly alleviate vacuolar degeneration of renal tubular epithelial cells and reduce interstitial edema. The high-dose group of CHF has the most significant effect (Figure 2G). To further quantify renal tubular injury, the tubular injury score was assessed (Figure 2H). The scoring results showed that CHF treatment markedly reduced the tubular injury score compared with the ATO group, indicating a significant amelioration of renal structural damage. The results of TEM showed that ATO exposure led to mitochondrial swelling, ridge rupture and excessive fragmentation in renal tubular epithelial cells. CHF treatment restored mitochondrial morphology, among which the high-dose group had a significant protective effect after treatment (Figure 2I). Based on these results, we selected the CHF-H group for subsequent mechanism studies to clarify the potential pathways of its renoprotective activity.

### 3.3 Transcriptomics demonstrated CHF targeted genes and signaling related to mitochondrial function, mitophagy and apoptosis

**FIGURE 3:**
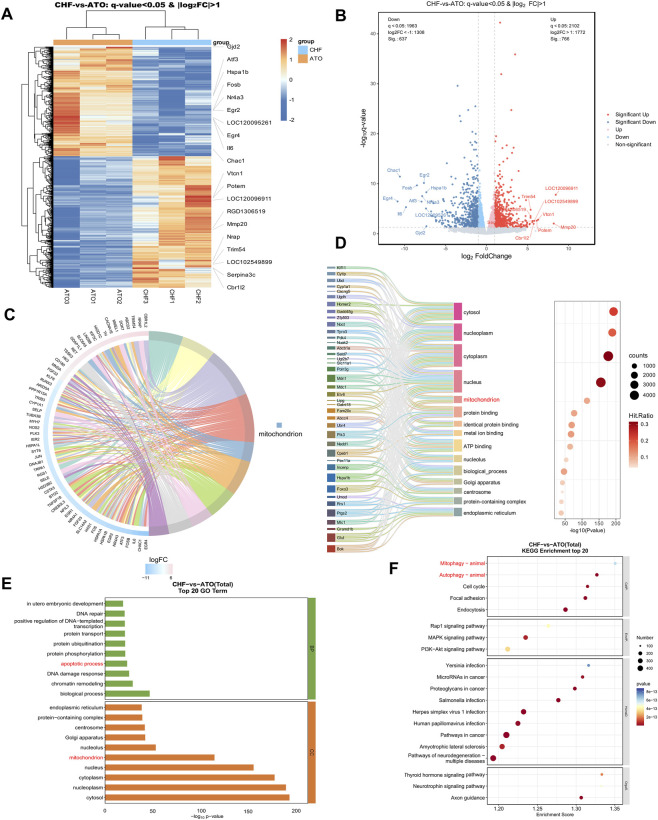
Transcriptomic analysis revealing CHF targeted genes and signaling pathways in AI-AKI rats. **(A)** Heat map of differentially expressed genes between the CHF and ATO groups. **(B)** Volcano plot of differentially expressed genes between the CHF and ATO groups. **(C, D)** The KEGG chord diagram and sankey bubble chart visualizes the relationships between genes and their enriched pathways between the CHF and ATO groups. **(E)** GO enrichment analysis of differentially expressed genes between the CHF and ATO groups. **(F)** The KEGG pathway enrichment bubble plot illustrates the enrichment of differentially expressed genes between the CHF and ATO groups.

To explore the renoprotective mechanism of CHF, transcriptomic analyses was performed to compare the ATO and CHF groups. A total of 1,403 differentially expressed genes (DEGs) were identified and visualized in the heatmap and volcano plots (Figures 3A,B). These DEGs between the ATO and CHF groups were subsequently subjected to clustering analysis, GO, and KEGG enrichment analyses to investigate the key signaling pathways. The KEGG chord diagram and sankey bubble chart visualizes the relationships between genes and their enriched pathways between the CHF and ATO groups (Figures 3C,D). The GO and KEGG enrichment analyses revealed the top 20 pathways, respectively (Figures 3E,F). The results indicated that the key signaling pathways involve mitochondrial function, mitophagy and apoptosis. These findings suggest that CHF may exert renoprotective effects by modulating MQC–related pathways, including mitophagy, while also influencing apoptotic signaling.

### 3.4 CHF protects against AI-AKI by modulating renal tubular epithelial cell MQC in rats

**FIGURE 4:**
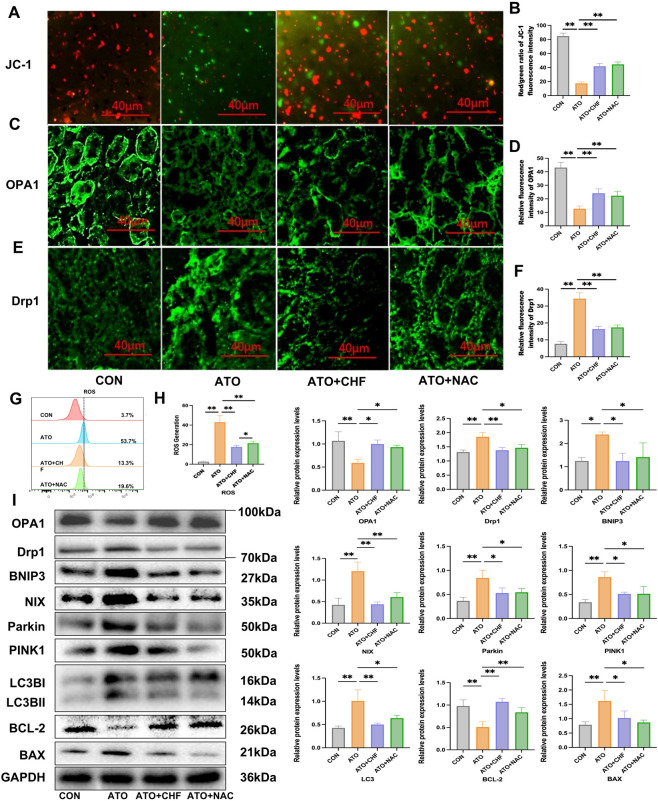
CHF protects against AI-AKI by modulating renal tubular epithelial cell MQC in rats. **(A, B)** Mitochondrial membrane potential (Δψm) was detected by JC-1 staining. **(C–F)** OPA1 and Drp1 expression was assessed by immunofluorescence (scale bar = 40 µm) in rat kidney tissues. **(G, H)** Flow cytometry results of mitochondria ROS in rat kidney tissues. **(I)** Western blotting analysis of OPA1, Drp1, BNIP3, NIX, Parkin, PINK1, LC3B, Bcl-2 and BAX. (p <0.05, *p <0.01 vs CON or ATO or NAC, n=3).

By extracting mitochondria from fresh kidney tissues of rats for JC-1 staining, the results showed that the JC-1 aggregate/monomer ratio in the ATO group was significantly decreased, and the CHF-H group could reverse this phenomenon and protect mitochondrial function (Figures 4A,B). Immunofluorescence staining of renal tissue sections revealed that ATO treatment would reduce the expression of OPA1 and increase the expression of Drp1, causing mitochondrial breakage. CHF, by up-regulating OPA1, reduced the level of Drp1 and preserved the structural integrity of mitochondria (Figures 4C–F).

The results of flow cytometry showed that ATO treatment significantly increased the mitochondrial ROS level in rat renal tissue, and CHF significantly reduced the production of ROS and decreased the occurrence of mitochondrial oxidative stress. Moreover, CHF reduced ATO-induced ROS production more effectively than NAC (Figures 4G,H). Western blot results demonstrated that ATO treatment significantly reduced the expression of fusion regulator OPA1, increased the expression of Drp1, BNIP3, NIX, Parkin, LC3B, and PINK1, upregulated the expression of pro-apoptotic protein BAX, and downregulated the expression of anti-apoptotic protein Bcl-2, indicating that mitochondria-mediated apoptosis was activated. CHF treatment reversed these changes by promoting OPA1 expression, reducing the levels of mitophagy-related proteins (Drp1, BNIP3, NIX, Parkin, LC3B, and PINK1), restoring the balance between BAX and Bcl-2, inhibiting mitochondrial apoptosis, and regulating MQC (Figure 4I). In conclusion, these findings suggest that ATO exposure induce mitochondrial dysfunction and mitochondrial oxidative stress in renal tissue, while CHF prevents AI-AKI by regulating MQC.

### 3.5 Effects of ATO on apoptosis, mitochondrial function, mitophagy related signaling, and mitochondrial dynamics *in vitro* with HK-2 cell line

**FIGURE 5:**
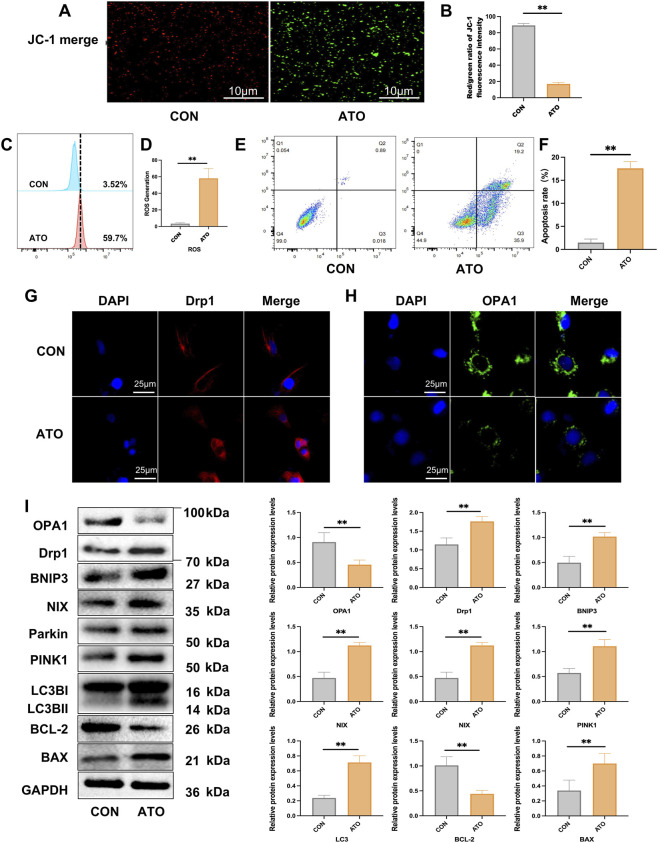
Effects of ATO on apoptosis, mitochondrial function, mitophagy related signaling, and mitochondrial dynamics *in vitro*. **(A, B)** Mitochondrial membrane potential (Δψm) was detected by JC-1 staining (scale bar = 10 µm) in HK-2 cells. **(C, D)** Flow cytometry results of mitochondria ROS in HK-2 cells. **(E, F)** Flow cytometry results of apoptosis in HK-2 cells. (*p < 0.05, **p < 0.01 vs. CON or ATO, n = 3). **(G, H)** Immunofluorescence image of Drp1 and OPA1 in HK-2 cells (scale bar = 25 µm). **(I)** Western blotting analysis of OPA1, Drp1, BNIP3, NIX, Parkin, PINK1, LC3B, Bcl-2 and BAX. (*p < 0.05, **p < 0.01 vs. CON or ATO, n = 3).

JC-1 staining of HK-2 cells showed that the red/green fluorescence ratio of HK-2 cells treated with ATO was significantly reduced, indicating a significant loss of mitochondrial membrane potential (Δψm) (Figures 5A,B). Flow cytometry analysis showed that ATO treatment significantly increased mitochondrial ROS levels in HK-2 cells (Figures 5C,D). Flow cytometry showed an increase in the proportion of apoptotic cells after ATO exposure (Figures 5E,F). Immunofluorescence showed that Drp1 expression was increased in ATO-treated cells, whereas OPA1 expression was decreased, indicating that ATO induced mitochondrial dysfunction (Figures 5G,H).

To further determine whether ATO affected the level of apoptosis and mitophagy of renal tubular epithelial cells, we examined the expression of key proteins related to mitophagy and apoptosis in HK-2 cell line by Western blot. The analysis revealed that ATO exposure resulted in the up-regulations of the mitochondrial fission related protein Drp1 and mitophagy markers PINK1, Parkin, BNIP3, NIX and LC3B, increased expression of the pro-apoptotic protein BAX, as well as decreased expression of the anti-apoptotic protein Bcl-2, which further confirming the activation of the mitochondrial apoptotic pathway (Figure 5I). Taken together, these results suggest that ATO induces mitochondrial dysfunction, excessive mitophagy and apoptosis in HK-2 cells.

## 4 Discussion

ATO exposure is recognized as a pivotal cause of AI-AKI. Recent studies have shown that prolonged ATO exposure not only triggers renal tubular injury but also impairs the regenerative capacity of tubular progenitor cells, leading to maladaptive repair (Haque et al., 2025). Such persistent defects in tubular regeneration underscore the broader pathological impact of arsenic toxicity and highlight the critical importance of maintaining mitochondrial integrity for effective renal recovery. Although mitochondrial injury is known to be an early event in arsenic nephrotoxicity, the specific mechanisms by which MQC becomes dysregulated have remained unclear (Liu et al., 2023). In the present study, we found that ATO evoked MQC, including OPA1- and Drp1-mediated mitochondrial dynamics and PINK1/Parkin-dependent mitophagy in renal tubular epithelial cells.

MQC plays a pivotal role in the pathogenesis and recovery of AKI. Accumulating evidence indicates that disruption of MQC contributes significantly to AKI development and hinders renal repair (Zhang et al., 2021). ATO the principal target cells of renal injury, tubular epithelial cells (TECs) are highly susceptible to damage due to their exposure to low oxygen tension, limited blood flow, and accumulation of toxic substances (Hall and de Seigneux, 2022). Among the underlying mechanisms, mitochondrial dysfunction resulting from impaired MQC has emerged as a central cause of TEC death (Tang et al., 2021). Recent studies have increasingly focused on targeting mitochondrial dysfunction as a therapeutic strategy for AKI (Yao et al., 2024; Yang et al., 2019; Li et al., 2018). MQC preserves mitochondrial homeostasis primarily through three processes: mitochondrial dynamics, mitophagy, and mitochondrial biogenesis (Ye et al., 2025). Among these, mitochondrial dynamics play a crucial role in promoting the repair of damaged TECs. Mitochondrial dynamics encompass two opposing but coordinated processes-fusion and fission (Jiang et al., 2022). Fusion involves the merging of the outer (OMM) and inner (IMM) mitochondrial membranes, allowing exchange of mitochondrial contents and stabilization of cristae structure (Cao et al., 2017). OMM fusion is mainly regulated by Mitofusin 1 (Mfn1) and Mitofusin 2 (Mfn2), whereas IMM fusion is mediated by OPA1 (Zhou et al., 2024). When OMM fusion proceeds without successful IMM fusion, the partially fused mitochondria are prone to subsequent fission and fragmentation (Mattie et al., 2019). Notably, OPA1-mediated IMM fusion serves as a critical determinant of mitochondrial structure integrity and functional stability. Fission, predominantly mediated by Drp1, facilitates the segregation of damaged mitochondrial segments for degradation via mitophagy (Zerihun et al., 2023).

OPA1 is a GTPase located on the inner mitochondrial membrane that governs inner membrane fusion and cristae integrity. Normal OPA1 is vital for maintaining mitochondrial membrane potential, ATP synthesis, and cell viability. Studies have demonstrated that OPA1 not only sustains mitochondrial morphology but also modulates apoptosis and autophagy by regulating cristae remodeling (Daumke and van der Laan, 2025). Loss or downregulation of OPA1 results in the collapse of cristae structure, dissipation of membrane potential, and metabolic dysfunction, leading to autophagic or apoptotic cell death. In ischemia-reperfusion AKI, oxidative stress markedly reduces OPA1 expression, exacerbating mitochondrial damage and tubular cell death. Conversely, maintaining or enhancing OPA1 expression alleviates mitochondrial fragmentation, decreases ROS accumulation, and attenuates renal tissue injury, highlighting its protective role in AKI (Xu et al., 2025).

Drp1, by contrast, mediates mitochondrial outer membrane fission. It assembles on mitochondrial surfaces into spiral structures and constricts them through GTP hydrolysis. Drp1 activity is tightly regulated by post-translational modifications such as phosphorylation, ubiquitination, and SUMOylation. Excessive activation of Drp1 leads to uncontrolled mitochondrial fragmentation, disrupted energy production, and ROS accumulation, promoting apoptosis and autophagy-associated cell death (Tábara et al., 2025). In AKI models, Drp1 expression and activation are markedly upregulated, correlating with mitochondrial fragmentation and tubular apoptosis, whereas Drp1 inhibition restores mitochondrial morphology, reduces oxidative stress, and mitigates renal damage (Pavlović et al., 2025).

Importantly, OPA1 and Drp1 act antagonistically to maintain mitochondrial dynamics. OPA1 promotes fusion, while Drp1 drives fission; together they preserve mitochondrial homeostasis. Studies have shown that in OPA1-deficient cells, additional Drp1 knockout can partially rescue cristae morphology and prevent mtDNA loss, suggesting that Drp1 deletion mitigates structural defects caused by OPA1 loss (Sessions et al., 2022). Thus, the interplay between OPA1 and Drp1 is fundamental to mitochondrial integrity and function.

Mitophagy is a critical mechanism for cells to maintain MQC and energy homeostasis, involving the recognition, encapsulation, transportation, and degradation of damaged mitochondria (Pan et al., 2025). When mitochondrial membrane potential decreases or oxidative stress intensifies, PINK1 accumulates on the outer membrane instead of being promptly imported into the inner membrane, recruiting E3 ubiquitin ligase Parkin. Activated Parkin ubiquitinates outer membrane proteins, marking mitochondria for autophagic degradation, which ultimately concludes through lysosomal enzymes in the mitophagy flux (Clague and Urbé, 2025). However, in AKI this flux is often blocked or abnormally activated, leading to the accumulation or excessive degradation of damaged mitochondria, thereby exacerbating cellular damage. Previous studies have shown that arsenic-induced blockage of this flux is a key mechanism in AKI, with restoring its integrity proving effective in ameliorating renal injury (Song et al., 2025b). At the molecular level, the PINK1/Parkin axis and BNIP3/NIX axis are crucial signaling pathways regulating mitophagy. The former is mainly activated after membrane potential loss, selectively clearing damaged mitochondria; the latter directly mediates the binding of damaged mitochondria to the autophagosome membrane (Clague and Urbé, 2025). Research has found that in contrast agent-induced AKI, receptor-mediated mitophagy via BNIP3/NIX is significantly enhanced, yet lysosomal dysfunction leads to blockages in the mitophagy flux, resulting in the accumulation of autophagosomes (Song et al., 2025b). Persistent mitochondrial damage further triggers mitophagy, a selective degradation process that eliminates dysfunctional mitochondria (Zhang et al., 2025). Although mitophagy is initially protective, its sustained activation can lead to mitochondrial depletion and aggravate tubular injury (Ai et al., 2025).

CHF contains several bioactive constituents such as ligustrazine, rhein, and berberine. These compounds have been reported to exert antioxidant, anti-inflammatory, and cytoprotective effects in various models of ischemic or toxic organ injury (Li et al., 2025; Yang et al., 2025; Fu et al., 2024). In this study, CHF treatment upregulated the mitochondrial fusion protein OPA1 and downregulated the fission protein Drp1, thereby restoring the balance between mitochondrial fusion and fission. ATO disrupts this balance, leading to excessive mitochondrial fragmentation, loss of membrane potential, and mitophagy, aggravating mitochondrial dysfunction. By modulating OPA1 and Drp1 expression, CHF helps maintain mitochondrial morphology and quality control. Tetramethylpyrazine (TMP), one of the major components of CHF has been shown to mitigate AI-AKI by reducing oxidative stress, suppressing inflammatory responses and modulating autophagy flux (Li and Gong, 2022; Song et al., 2025b). Based on the previously published clinical research data, CHF can partially promote renal function recovery in AKI patients by enhancing the HO-1-mediated antioxidant defense system and suppressing inflammatory pathways involving NLRP3, IL-6, and CCL2 (Gong et al., 2020; Gong et al., 2021; Song and Gong, 2024). Considering the pivotal role of mitochondrial homeostasis in AKI pathogenesis, clarifying how CHF regulates mitochondrial fusion-fission balance and mitophagy holds both mechanistic and therapeutic importance.

Mitochondrial dysfunction impairs cellular energy metabolism and serves as a major source of ROS, which trigger oxidative stress and destabilize mitochondrial DNA (mtDNA). Moreover, efficient ROS scavenging is essential for maintaining mitochondrial function and cellular integrity (Lennicke and Cochemé, 2021). As a classical antioxidant drug in clinical practice, NAC can increase GSH levels, eliminate ROS, maintain mitochondrial membrane potential, and alleviate mitochondrial dysfunction caused by oxidative stress. NAC showed effective renoprotective effects against AI-AKI, which further confirmed the crucial role of oxidative stress in AI-AKI pathological process. In the present study, we demonstrated that ATO exposure with clinical related dose should evokes renal tubular epithelial cell MQC, including alterations in mitochondrial dynamics and mitophagy. Importantly, CHF exerts renoprotective effects by restoring mitochondrial homeostasis through the regulation of key mitochondrial dynamics proteins, thereby preventing pathological mitochondrial fragmentation and mitophagy of renal tubular epithelial cells.

## 5 Conclusion

**FIGURE 6:**
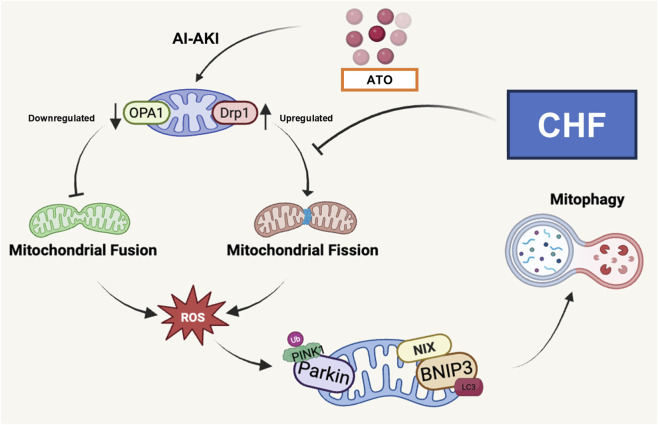
Mechanism diagram of CHF against AI-AKI by regulating the OPA1- and Drp1-mediated mitochondrial dynamics imbalance and mitophagy in renal tubular epithelial cells.

In summary, the pathogenesis of AI-AKI involves mitochondrial oxidative stress, OPA1- and Drp1-mediated mitochondrial dynamics imbalance, and PINK1/Parkin-dependent mitophagy in renal tubular epithelial cells both *in vivo* and *in vitro* (Figure 6). CHF could attenuates such a renal injury by restoring mitochondrial quality control and preserving mitochondrial homeostasis, which highlighting its therapeutic potential against AI-AKI.

## Data Availability

The datasets presented in this study can be found in online repositories. The names of the repository/repositories and accession number(s) can be found below: https://figshare.com/, https://figshare.com/s/1e2477ad40d08c44c386 (DOI: 10.6084/m9.figshare.30451286).
