# Supplementary figures and images for: Arsenic trioxide-induced acute kidney injury: OPA1- and Drp1-mediated mitochondrial dynamics imbalance, PINK1/Parkin-dependent mitophagy, and Chuanhuang Fang III
Source: Front Mol Biosci. 2026 Jan 30;13:1778855. doi: 10.3389/fmolb.2026.1778855 (PMC12900759; doi:10.3389/fmolb.2026.1778855)

## Original Western blot data

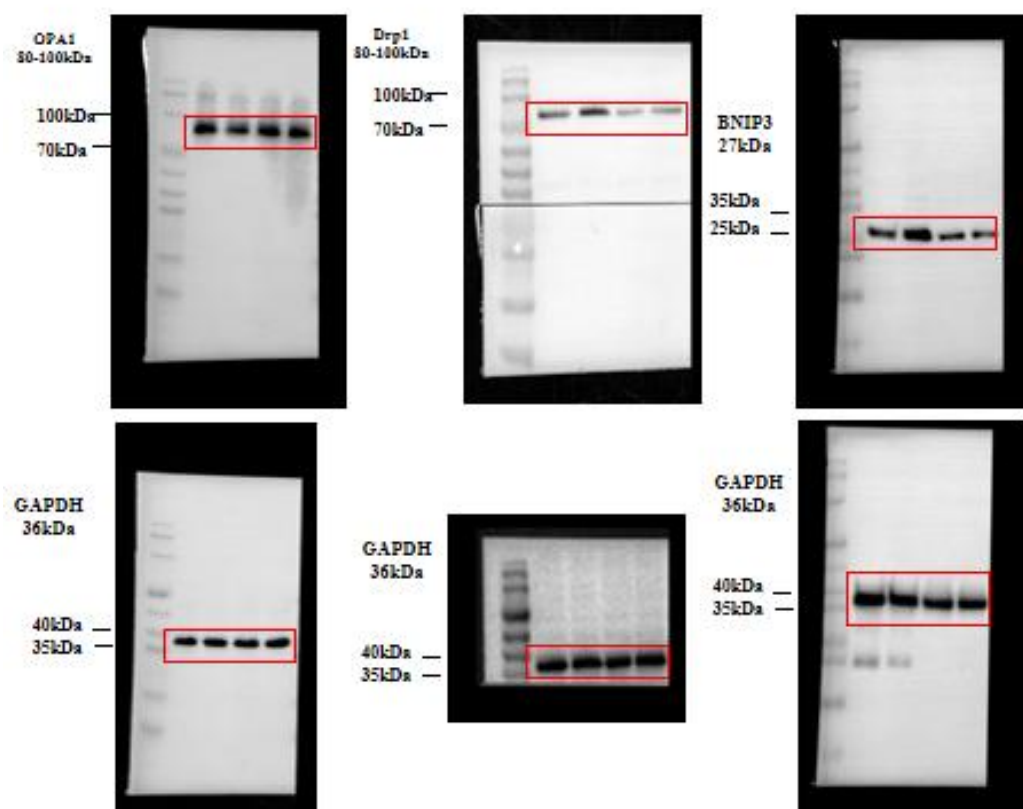

Figure.4I

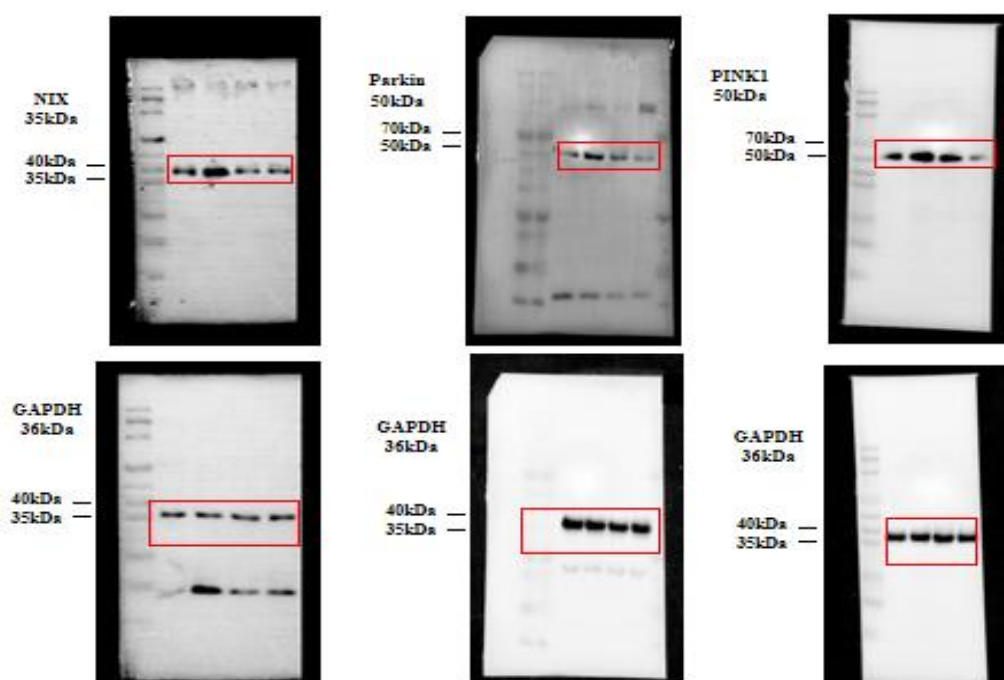

Figure.4I

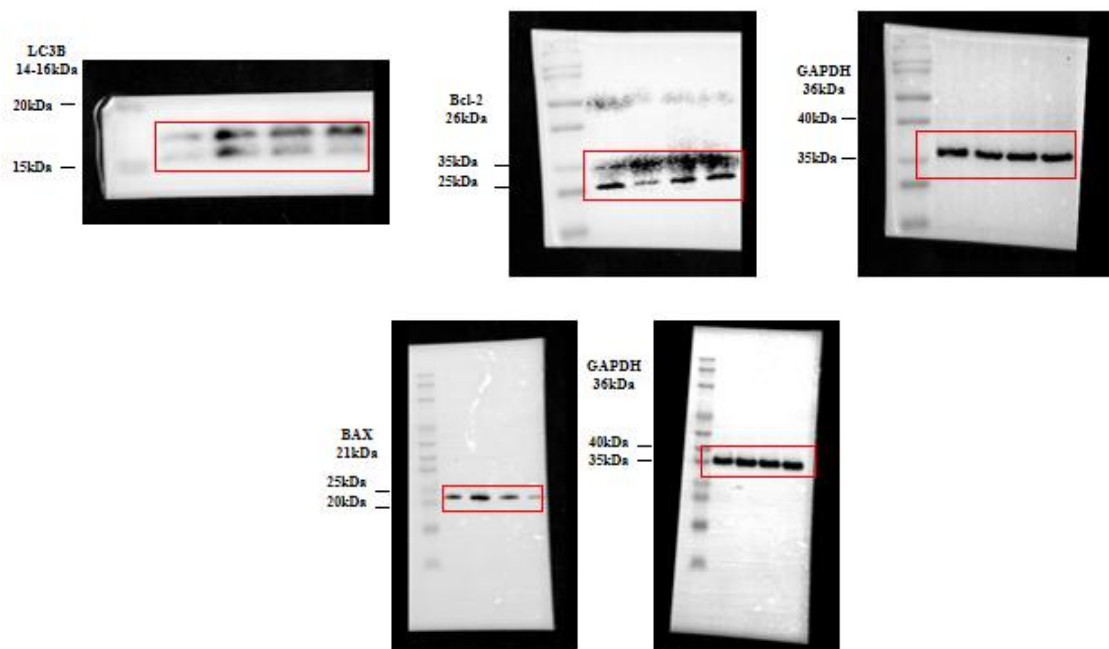

Figure.4I

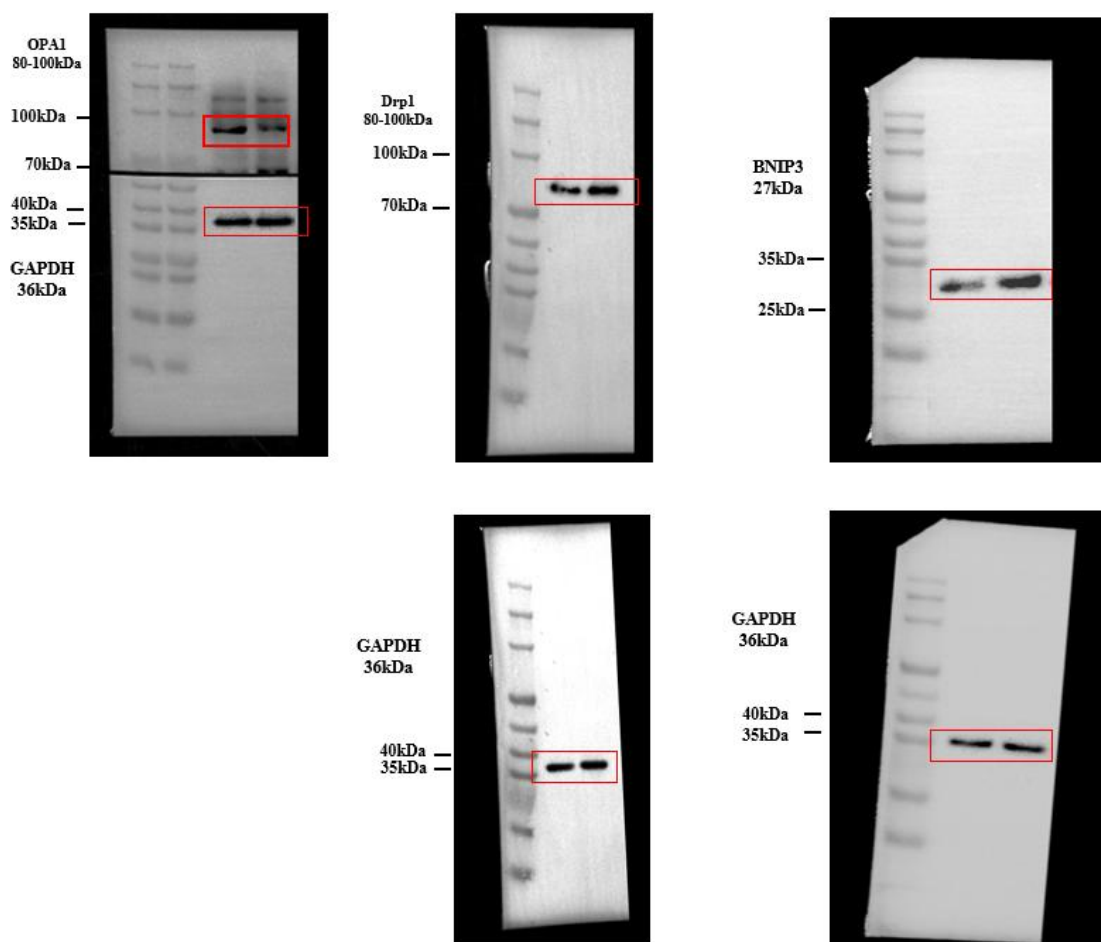

Figure.5I

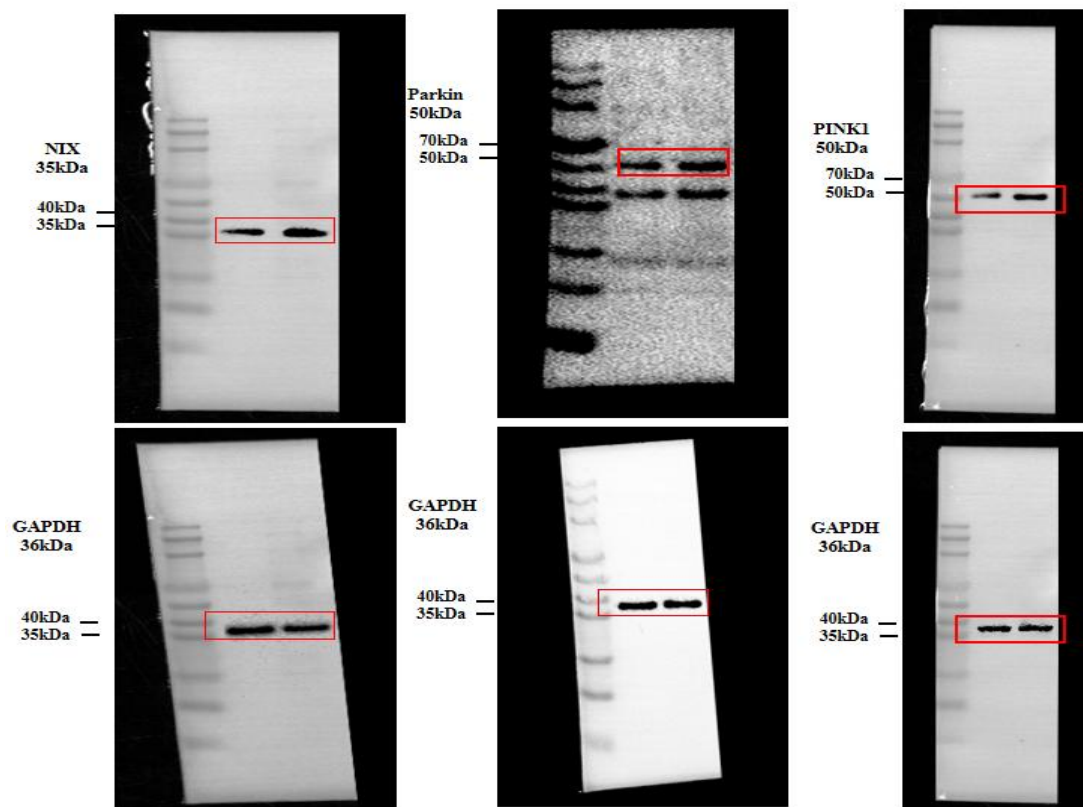

Figure.5I

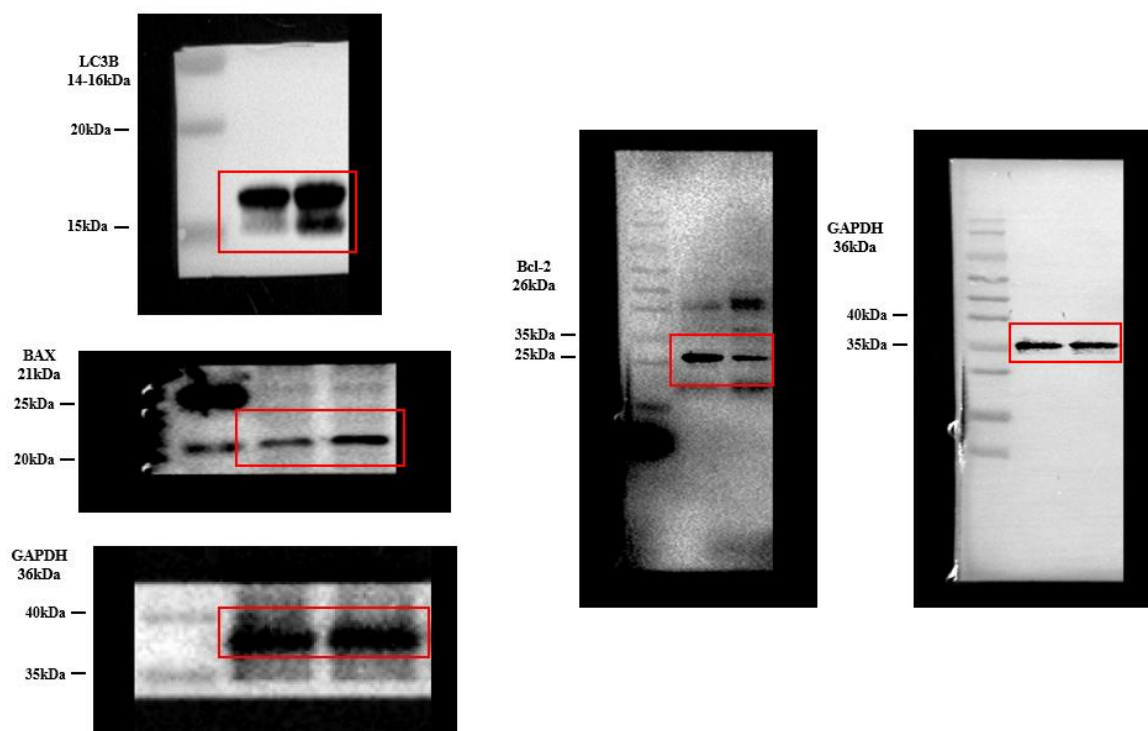

Figure.5I

Supplement: Supplementary file 1 [file DataSheet1.pdf]
